# Carotid Perivascular Adipose Tissue Density as a Marker of Large Artery Atherosclerotic Stroke in Patients Undergoing Mechanical Thrombectomy for Acute Middle Cerebral Artery Occlusion

**DOI:** 10.3390/jcm15114369

**Published:** 2026-06-05

**Authors:** Samet Genez, Sümeyra Nur Atasoy, Umit Mustak, Hamza Özer, Yunus Yılmazsoy, Muhammed Nur Öğün, Hilmiye Tokmak, Murat Yılmaz, Sadettin Ersoy

**Affiliations:** 1 Department of Radiology, Faculty of Medicine, Bolu Abant Izzet Baysal University, Bolu 14030, Turkey; 2 Department of Radiology, Faculty of Medicine, Sakarya University, Sakarya 54100, Turkey; 3 General Directorate of Public Health, Ministry of Health, Ankara 06800, Turkey; 4 Department of Neurology, Niğde Training and Research Hospital, Niğde 51100, Turkey; 5 Department of Neurology, Faculty of Medicine, Bolu Abant Izzet Baysal University, Bolu 14030, Turkey

**Keywords:** acute ischemic stroke, mechanical thrombectomy, carotid PVAT density, computed tomography angiography, stroke etiology, large artery atherosclerosis

## Abstract

**Background/Objectives**: Carotid perivascular adipose tissue (PVAT) density on computed tomography angiography (CTA) is a noninvasive surrogate marker of local vascular inflammation, but its relevance to stroke etiology in a homogeneous cohort of patients undergoing mechanical thrombectomy (MT) remains unclear. **Methods**: We retrospectively analyzed 146 consecutive patients with acute ischemic stroke treated with MT for acute middle cerebral artery (MCA) occlusion between May 2018 and August 2024. Baseline CTA was used to quantify carotid PVAT density with two 2–3 mm^2^ circular regions of interest per internal carotid artery (ICA), placed ≥1 mm from the vessel wall. Measurements were performed bilaterally, and the ICA ipsilateral to the occluded MCA was defined as the stroke-side ICA. Etiology was classified according to the Trial of ORG 10172 in Acute Stroke Treatment (TOAST) system and grouped as large-artery atherosclerosis (LAA), cardioembolism (CE), and other/undetermined (OD/UD). Interobserver agreement was assessed using the intraclass correlation coefficient. **Results**: The mean age was 72.21 ± 12.39 years; 83.6% of patients achieved successful recanalization (mTICI ≥ 2b), and 47.9% had a favorable 90-day outcome (mRS ≤ 2). In the LAA subgroup (*n* = 38), ipsilateral PVAT density was significantly higher (less negative) than contralateral PVAT density (−64.24 ± 11.74 vs. −78.22 ± 9.13 HU; *p* < 0.001). Ipsilateral PVAT density differed significantly across TOAST groups (ANOVA *p* = 0.004), being higher in LAA than in CE (Δ = 11.19 HU; *p* = 0.003) and OD/UD (Δ = 9.54 HU; *p* = 0.004). ROC analysis showed modest discrimination for LAA versus non-LAA stroke (AUC 0.67, 95% CI 0.58–0.75), with an optimal cutoff of −79 HU (sensitivity 92.1%, specificity 40.7%). In multivariable logistic regression, higher ipsilateral PVAT density was independently associated with LAA etiology (per 1-HU increase: OR 1.048, 95% CI 1.018–1.079; *p* = 0.0016). PVAT density was not associated with recanalization success or 90-day functional outcome. **Conclusions**: In patients with acute MCA occlusion undergoing MT, higher carotid PVAT density on the stroke side was independently associated with LAA stroke etiology but had limited value for predicting MT success or short-term clinical outcome.

## 1. Introduction

Acute ischemic stroke (AIS) remains a major global health concern, representing one of the leading causes of long-term disability and mortality worldwide [1,2]. For patients with acute large vessel occlusion (LVO), mechanical thrombectomy (MT) has become the standard of care and achieves high rates of angiographic reperfusion [3]. Despite high rates of reperfusion after MT, functional outcomes remain suboptimal, suggesting that underlying vascular and inflammatory pathology may influence recovery [4].

Perivascular adipose tissue (PVAT) is a metabolically active fat depot that surrounds blood vessels and communicates directly with the vascular wall without a distinct anatomic barrier [5]. In the setting of vascular inflammation, PVAT undergoes compositional changes, including reduced lipid and increased water content, which result in higher computed tomography (CT) attenuation. This imaging characteristic reflects local vascular inflammation and can be quantitatively assessed on CT or computed tomography angiography (CTA) [6,7,8].

Around the carotid arteries, elevated PVAT density has been consistently associated with carotid atherosclerosis, plaque vulnerability, and cerebrovascular ischemic events [9,10]. Imaging and histopathologic studies have demonstrated increased PVAT density around the stenotic internal carotid artery (ICA), which correlates with features of vulnerable plaque such as intraplaque hemorrhage, fibrous cap rupture, and advanced American Heart Association (AHA) type VI lesions [11,12,13]. These observations support the concept that carotid PVAT density captures the inflammatory component of atherosclerotic disease beyond luminal stenosis alone.

Recently, PVAT density has also been investigated in the context of acute stroke treatment. In patients receiving intravenous thrombolysis, higher PVAT density has been reported in association with poorer functional outcomes [14]. Increased carotid PVAT density has also been linked to unsuccessful recanalization and higher mortality [15]. However, data on PVAT density in homogeneous MT cohorts with acute middle cerebral artery (MCA) occlusion are limited, and the relationship between carotid PVAT density and stroke etiology, particularly large artery atherosclerosis (LAA), has not been systematically evaluated.

In the present study, we focused on isolated MCA-M1 occlusions to provide a relatively homogeneous anterior-circulation MT cohort and to minimize the anatomic and etiologic heterogeneity associated with other anterior-circulation occlusion patterns, such as ICA or tandem occlusions. The primary objective was to determine whether increased ipsilateral carotid PVAT density, as a potential imaging marker of local vascular inflammation, is associated with LAA stroke in patients undergoing MT. We also explored the relationship between carotid PVAT density, recanalization success, and 3-month functional outcomes.

## 2. Materials and Methods

This single-center retrospective study included patients with acute ischemic stroke who underwent MT between May 2018 and August 2024. The study was approved by the institutional ethics committee and conducted in accordance with the Declaration of Helsinki. Written informed consent was waived due to the retrospective nature of the study.

### 2.1. Patient Selection

Inclusion criteria were: (1) age ≥ 18 years; (2) anterior circulation LVO involving the M1 segment of the MCA treated with MT; (3) availability of baseline CTA before the procedure; (4) documentation of the final angiographic result after MT; and (5) baseline National Institutes of Health Stroke Scale (NIHSS) ≥ 6 and Alberta Stroke Program Early CT Score (ASPECTS) ≥ 6, consistent with institutional eligibility criteria for thrombectomy. Exclusion criteria were posterior-circulation stroke, tandem cervical ICA–intracranial occlusion, MCA occlusion primarily attributable to intracranial atherosclerotic disease, missing or poor-quality baseline CTA, and missing clinical or imaging outcome data. Non-tandem ICA occlusions were also excluded to preserve a homogeneous MCA-M1 thrombectomy cohort, as most ICA occlusions in our institutional cohort were tandem lesions, and the remaining isolated ICA occlusions were too few for reliable subgroup analysis.

### 2.2. Endovascular Procedure and Clinical Data

All procedures were performed by three neurointerventionalists, each with more than 7 years of experience, using stent retrievers, aspiration catheters, or combined techniques. Conscious sedation was used as the default anesthetic approach, with general anesthesia applied when clinically required. Procedural details, including onset-to-groin puncture time, procedure duration, number of device passes, and periprocedural complications (distal embolization, vessel perforation, or dissection) were recorded. The degree of reperfusion was graded using the modified Thrombolysis in Cerebral Infarction (mTICI) score, and successful recanalization was defined as mTICI 2b–3 on the final angiogram.

### 2.3. Clinical and Etiologic Assessment

Stroke etiology was initially classified according to the Trial of Org 10172 in Acute Stroke Treatment (TOAST) criteria by a stroke neurologist on available clinical information, vascular imaging, and cardiac evaluation [16]. For statistical analysis, etiologies were grouped into three categories: large artery atherosclerosis (LAA), cardioembolism (CE), and other determined or undetermined etiologies (OD/UD). LAA etiology was assigned according to TOAST criteria when vascular imaging demonstrated >50% stenosis in an ipsilateral extracranial carotid artery, and no competing high-risk cardiac or other plausible stroke mechanism was identified. CE etiology was assigned when a high-risk cardiac source, such as atrial fibrillation, was documented and no competing large-artery or other plausible stroke mechanism was identified. OD/UD etiology included patients with other determined causes, incomplete or negative standard etiologic evaluation, or multiple competing potential etiologies, including atrial fibrillation together with significant ipsilateral large-artery atherosclerosis.

Demographic data and vascular risk factors, including hypertension, diabetes mellitus, atrial fibrillation, smoking, coronary artery disease, and dyslipidemia, were collected from the hospital database. Stroke severity and early ischemic changes were assessed using baseline NIHSS and ASPECTS scores, respectively. Functional outcome was evaluated at 90 days using the modified Rankin Scale (mRS), with an mRS score ≤ 2 indicating a favorable outcome.

### 2.4. CTA Acquisition and PVAT Density Measurement

All patients underwent baseline CTA in the angiographic phase after intravenous administration of iodinated contrast medium (Omnipaque 350, 1 mL/kg at 4.5 mL/s) using a 64-slice multidetector CT scanner (Revolution EVO, GE Healthcare, Waukesha, WI, USA). Bolus tracking was initiated when the attenuation in the cervical ICA reached 100 Hounsfield units (HU). Standard acquisition parameters were applied (tube voltage 100–120 kVp, tube current 200–350 mAs, slice thickness 0.5–1 mm).

**Figure 1:**
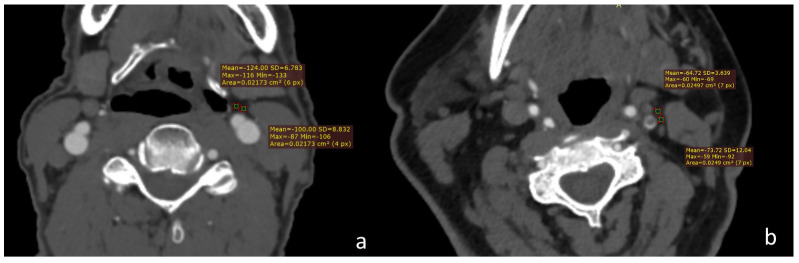
Representative baseline CTA images illustrating carotid perivascular adipose tissue (PVAT) density measurement. (**a**) PVAT density measurement on an internal carotid artery (ICA) without visible carotid plaque/stenosis, with ROIs placed at the carotid bulb/bifurcation level. (**b**) PVAT density measurement in a patient with significant ICA stenosis, with ROIs placed at the NASCET-defined level of maximal stenosis.

PVAT density was measured on baseline CTA following previously described methods for carotid PVAT quantification [9,14,15]. For each patient, the site and degree of maximal luminal narrowing for both ICAs were determined using the North American Symptomatic Carotid Endarterectomy Trial (NASCET) method. PVAT density measurements were performed on axial images using predefined display settings (window width 500 HU, window level 100 HU) as described in prior studies. For each ICA, two circular regions of interest (ROIs) of approximately 2–3 mm^2^ were manually drawn within the pericarotid fat, maintaining at least 1 mm distance from the arterial wall to avoid inclusion of the vessel wall, plaque/calcifications, and partial-volume effects. When a stenotic lesion was present, ROIs were positioned at the level of NASCET-defined maximal stenosis. If no visible stenosis was identified, ROIs were placed in the perivascular fat at the level of the carotid bulb/bifurcation (Figure 1). The ICA ipsilateral to the occluded MCA was defined as the stroke side, and the contralateral ICA served as the reference side. Two HU values were obtained for each ICA (ROI 1 and ROI 2), and the mean of these values represented the carotid PVAT density for that artery. PVAT measurements were independently performed by two radiologists, both blinded to clinical data, etiology assignment, and outcomes. Interobserver agreement for PVAT measurements was excellent, with an intraclass correlation coefficient (ICC) of 0.92 (95% CI, 0.88–0.95), indicating high reproducibility of the ROI-based measurement technique. For statistical analysis, the PVAT values from the two radiologists were averaged for each side.

### 2.5. Statistical Analysis

All data were analyzed using SPSS 22.0 (IBM Corp., Armonk, NY, USA). Normal distribution of the data was assessed using the Kolmogorov–Smirnov and Shapiro–Wilk tests. Normally distributed continuous variables were expressed as mean ± standard deviation, and non-normally distributed variables (if any) were expressed as median (minimum–maximum). Categorical variables are presented as numbers and percentages (%). PVAT values appeared approximately normally distributed based on visual inspection and Shapiro–Wilk testing; therefore, they were analyzed using parametric tests.

Comparisons between stroke-side and contralateral PVAT values were analyzed using a paired-samples *t*-test. The mean difference, 95% confidence interval (CI), and Cohen’s d effect size were also calculated for this analysis. One-way ANOVA was used to compare ipsilateral PVAT density across etiological subgroups (TOAST classification: LAA, CE, and OD/UD). Pairwise group comparisons were performed using Bonferroni-corrected post hoc tests, and Cohen’s d was calculated as a measure of between-group effect size. Independent-samples *t*-tests were used to compare PVAT values according to recanalization status (final mTICI < 2b vs. ≥2b), 3-month functional outcome (mRS > 2 vs. ≤2), and mortality (mRS = 6 vs. <6).

Logistic regression analyses were performed to evaluate the association of ipsilateral PVAT density with stroke etiology and clinical outcomes. For the etiology analysis, stroke subtype was dichotomized as LAA versus non-LAA, with non-LAA defined as CE and OD/UD etiologies. Univariable and multivariable logistic regression models were used to assess the association between ipsilateral PVAT density and LAA stroke, with ipsilateral PVAT density entered as a continuous predictor per 1-HU increase. Covariates for multivariable adjustment were selected a priori based on clinical relevance and prior literature. The multivariable model for LAA versus non-LAA etiology included age, sex, atrial fibrillation, hypertension, diabetes mellitus, smoking, and dyslipidemia. For successful recanalization and 90-day functional outcome, the multivariable models included ipsilateral PVAT density, age, baseline NIHSS, baseline ASPECTS, onset-to-groin puncture time, and atrial fibrillation. To minimize overfitting, the number of covariates was restricted according to the number of events. Ninety-day mortality was analyzed using univariable logistic regression only. Multicollinearity was assessed using variance inflation factors, and no problematic collinearity was identified. The discriminative ability of ipsilateral PVAT density for identifying LAA stroke was further assessed using ROC curve analysis, with calculation of the AUC and its 95% confidence interval. The optimal cutoff value was determined using the Youden index. Odds ratios with 95% confidence intervals were reported, and a two-sided *p* value < 0.05 was considered statistically significant.

## 3. Results

### 3.1. Baseline Characteristics

**Figure 2:**
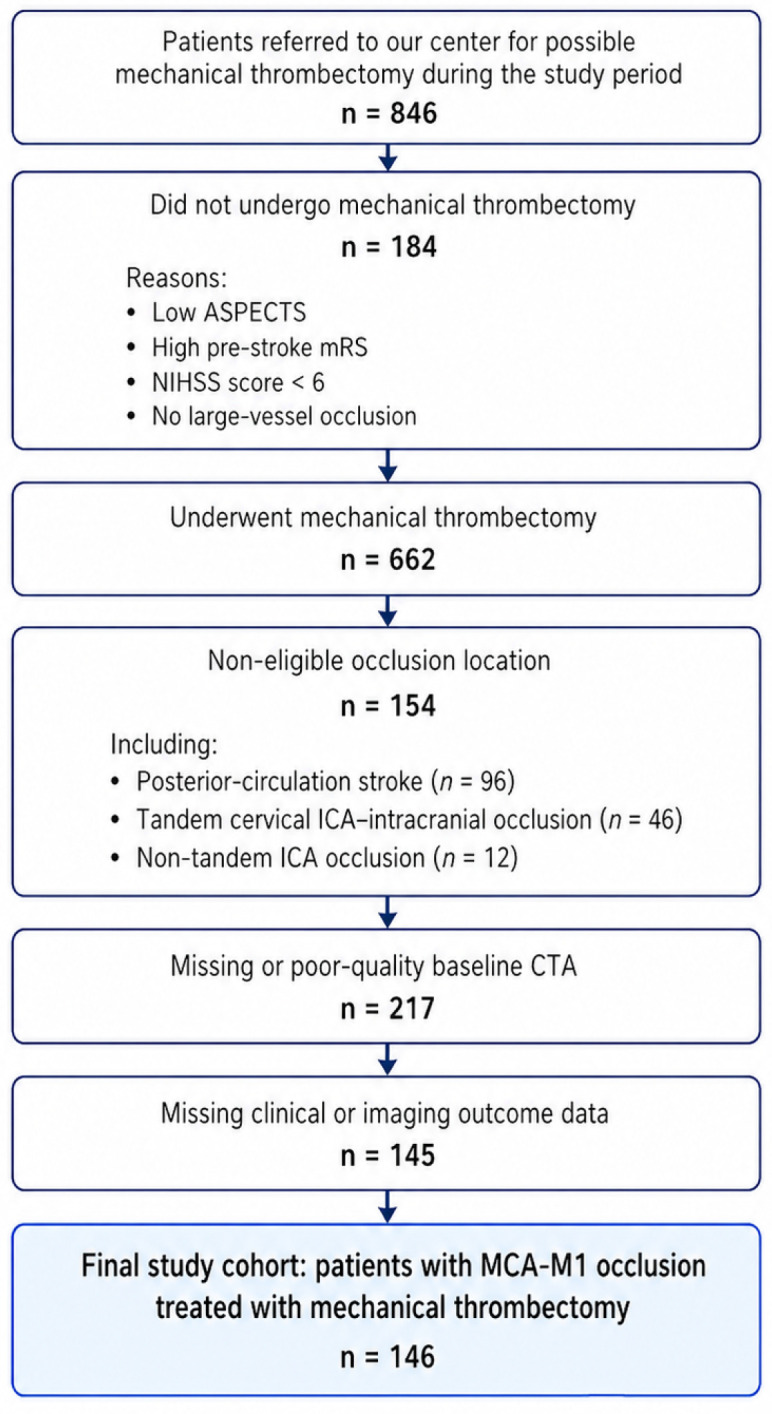
Flow diagram of patient selection.

Patient selection is summarized in Figure 2. During the study period, 846 patients referred for possible mechanical thrombectomy were screened. After applying the predefined exclusion criteria, the final study cohort consisted of 146 patients with MCA-M1 occlusion treated with MT. The mean age was 72.21 ± 12.39 years, and 82 patients (56.2%) were male. Vascular risk factors were frequent: hypertension was present in 92 patients (63.0%), diabetes mellitus in 40 (27.4%), dyslipidemia in 14 (9.6%), coronary artery disease in 51 (34.9%), and atrial fibrillation in 73 (50.0%). Thirty-six patients (24.7%) were active smokers.

The mean baseline NIHSS score was 12.81 ± 4.78, and the mean ASPECTS was 8.21 ± 1.44. Aspiration alone was used in 23 procedures (15.8%), whereas a combined technique (aspiration plus stent retriever) was used in 123 (84.2%). Successful recanalization (final mTICI ≥ 2b) was achieved in 122 patients (83.6%). Carotid stenting or carotid balloon angioplasty was performed in 15 cases (10.3%). All 15 patients had >70% ipsilateral extracranial ICA stenosis identified during the procedure. Carotid stenting was performed in 11 patients who were already receiving antiplatelet therapy, whereas balloon angioplasty alone was performed in the remaining 4 patients. At 90 days, poor functional outcome (mRS > 2) was observed in 76 patients (52.1%), favorable outcome (mRS ≤ 2) in 70 patients (47.9%), and mortality occurred in 29 patients (19.9%) (Table 1).

### 3.2. Ipsilateral and Contralateral PVAT Density in LAA Patients

In the LAA subgroup (*n* = 38), ipsilateral carotid PVAT density was higher (less negative) than contralateral PVAT density (−64.24 ± 11.74 HU vs. −78.22 ± 9.13 HU; Table 2). The mean paired difference was 13.99 HU (95% CI, 9.15–18.82 HU), with a large effect size (Cohen’s d = 0.95). This difference was statistically significant (t = 5.86, *p* < 0.001), indicating higher PVAT density on the stroke side in LAA patients.

### 3.3. PVAT Density According to TOAST Etiology

**Figure 3:**
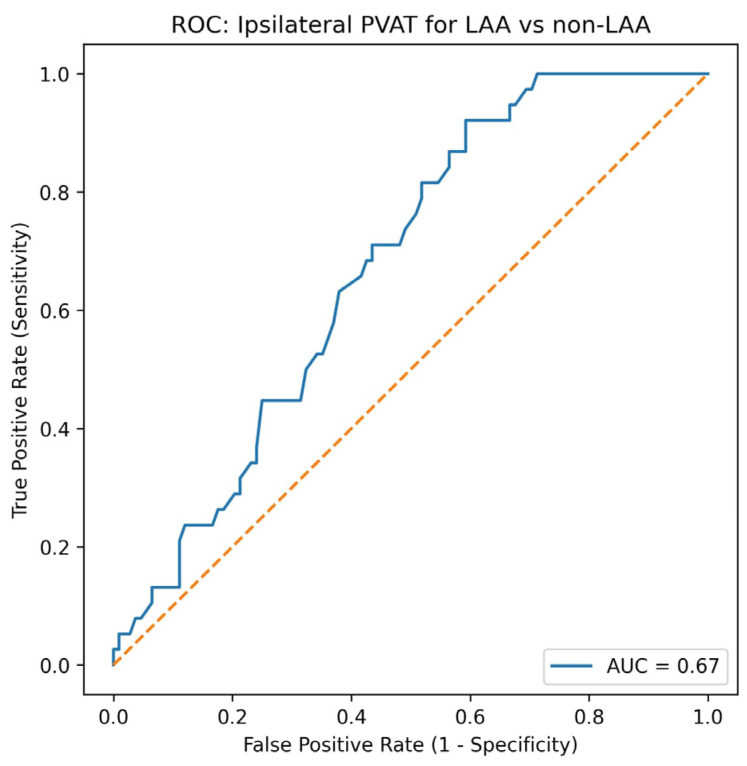
Receiver operating characteristic (ROC) curve of ipsilateral carotid PVAT density for discriminating large artery atherosclerosis (LAA) from non-LAA etiologies. The AUC was 0.67 (95% CI, 0.58–0.75). The optimal cutoff by the Youden index was −79 HU (sensitivity 92.1%, specificity 40.7%).

Ipsilateral PVAT density differed significantly across TOAST etiologic subgroups (ANOVA F = 5.72, *p* = 0.004; Table 2). Mean ipsilateral PVAT values were −64.24 ± 11.74 HU in the LAA group (*n* = 38), −75.42 ± 20.11 HU in the CE group (*n* = 58), and −73.78 ± 14.93 HU in the OD/UD group (*n* = 50). Bonferroni-corrected post hoc analyses showed higher ipsilateral PVAT density in LAA than in CE (mean difference 11.19 HU; adjusted *p* = 0.003; Cohen’s d ≈ 0.65) and higher PVAT density in LAA than in OD/UD (mean difference 9.54 HU; adjusted *p* = 0.004; Cohen’s d ≈ 0.70). No significant difference was found between CE and OD/UD groups. In the subgroup of 15 patients who underwent carotid stenting or carotid balloon angioplasty, ipsilateral carotid PVAT density was higher than contralateral PVAT density in a paired comparison (−66.8 ± 12.4 HU vs. −78.6 ± 8.5 HU; *p* = 0.017). In ROC analysis, ipsilateral PVAT density showed modest ability to discriminate LAA from non-LAA etiologies (AUC = 0.67; 95% CI, 0.58–0.75) (Figure 3). A threshold of −79 HU provided the highest Youden index, yielding a sensitivity of 92.1% and a specificity of 40.7% for identifying LAA strokes. In logistic regression analyses, including a binary logistic regression model of all patients, higher ipsilateral PVAT density was associated with increased odds of LAA stroke in both univariable and clinically adjusted multivariable models (Table 3).

### 3.4. PVAT Density and Recanalization Success

When patients were stratified according to angiographic recanalization status, ipsilateral PVAT density was −75.96 ± 16.87 HU in those with unsuccessful recanalization (final mTICI < 2b, *n* = 24) and −71.16 ± 17.06 HU in those with successful recanalization (final mTICI ≥ 2b, *n* = 122) (Table 2). The mean difference between groups was −4.80 HU (95% CI, −12.48 to 2.88 HU), with a small effect size (Cohen’s d ≈ 0.28), and was not statistically significant (*p* = 0.21). In multivariable logistic regression adjusted for age, baseline NIHSS, baseline ASPECTS, onset-to-groin puncture time, and atrial fibrillation, ipsilateral PVAT density was not independently associated with successful recanalization (per 1-HU increase: OR 1.02; 95% CI, 0.99–1.04; *p* = 0.24).

### 3.5. PVAT Density and 3-Month Functional Outcome

Ipsilateral PVAT values were similar between the 3-month outcome groups. PVAT density was −71.97 ± 17.84 HU in patients with poor functional outcome (mRS > 2, *n* = 76) and −71.92 ± 16.31 HU in those with favorable outcome (mRS ≤ 2, *n* = 70) (Table 2). The mean difference was −0.05 HU (95% CI, −5.64 to 5.53 HU), with an effect size close to zero (Cohen’s d ≈ −0.003), and no statistically significant difference was observed (*p* = 0.99). In multivariable logistic regression adjusted for age, baseline NIHSS, baseline ASPECTS, onset-to-groin puncture time, and atrial fibrillation, ipsilateral PVAT density was not independently associated with 3-month functional outcome (per 1-HU increase: OR 1.01; 95% CI, 0.98–1.03; *p* = 0.54). Older age, higher baseline NIHSS, and lower baseline ASPECTS were independently associated with 3-month outcome.

### 3.6. PVAT Density and 90-Day Mortality

Ninety-day mortality (mRS = 6) occurred in 29 patients (19.9%). In exploratory analyses, ipsilateral PVAT did not differ between patients who died and those who survived (−72.83 ± 15.91 HU vs. −71.73 ± 17.40 HU; *p* = 0.746) and was not associated with 90-day mortality in univariable logistic regression (OR per 1-HU increase: 0.996; 95% CI, 0.973–1.020; *p* = 0.756).

## 4. Discussion

This study investigated the association of carotid PVAT density with stroke etiology, recanalization success, and functional outcome in patients undergoing mechanical thrombectomy for acute MCA-M1 occlusion. Ipsilateral carotid PVAT density was higher in patients with LAA stroke than in those with CE or OD/UD etiologies. In contrast, PVAT density was not associated with successful recanalization or 90-day functional outcome in either univariable or multivariable analyses. Overall, these findings suggest that carotid PVAT density may reflect local atherosclerotic inflammation associated with LAA stroke, whereas its prognostic value for clinical outcomes after mechanical thrombectomy appears limited.

Our results are consistent with studies reporting that PVAT density reflects local vascular inflammation and plaque vulnerability [17]. Experimental and imaging studies have shown that inflammatory activity in the arterial wall alters the composition of adjacent perivascular fat, reducing lipid content and increasing water content, which leads to higher HU values on CT [7,18]. Clinical studies of carotid disease have consistently demonstrated higher carotid PVAT density around vulnerable or symptomatic plaques, particularly those with intraplaque hemorrhage, fibrous cap rupture, or AHA type VI morphology [11,13]. Kashiwazaki et al. further linked higher carotid PVAT density to positive remodeling and to higher macrophage and microvessel counts on histology, directly supporting PVAT density as a tissue marker of plaque inflammation [19]. These findings support increased carotid PVAT density as a marker of local plaque inflammation and vulnerability in patients with LAA stroke [15,20]. In exploratory analyses, ipsilateral PVAT density showed only a weak correlation with ipsilateral NASCET stenosis in the overall cohort and no significant correlation within etiologic subgroups, suggesting that PVAT density may capture inflammatory information beyond luminal narrowing alone [9,19] (Table S1).

A critical consideration in interpreting our findings is the biological link between pericarotid inflammation and anatomically distinct MCA occlusion. A plausible mechanistic explanation is artery-to-artery embolism, a well-recognized mechanism of LAA stroke. Although the cervical ICA and the MCA are anatomically distinct vascular segments, carotid PVAT density may serve as a surrogate imaging marker of inflammatory activity and plaque vulnerability in the upstream cervical ICA rather than reflecting local MCA wall inflammation itself. Inflammatory changes within an unstable carotid plaque may increase the CT attenuation of the surrounding perivascular adipose tissue. Increased carotid PVAT density has also been associated with high-risk plaque features, including intraplaque hemorrhage, lipid-rich necrotic core, and fibrous cap disruption. In this context, an inflamed and vulnerable cervical ICA plaque may act as an upstream embolic source for downstream MCA-M1 occlusion.

In cohorts enriched for carotid stenosis, PVAT density has also been associated with prevalent or prior ischemic events and with symptomatic versus asymptomatic plaques. Baradaran et al. showed that higher PVAT density is associated with cerebrovascular ischemic events independent of stenosis degree [9]. Besler et al. and Zhang et al. reported that higher PVAT density predicts symptomatic carotid disease with AUC values of 0.75–0.81 and odds ratios around 1.04–1.14 per 1-HU increase [8,10]. Taken together, these studies are consistent with our finding that ipsilateral PVAT density is highest in LAA strokes.

Our data on MT outcomes differ from those of Jin et al., who reported that higher carotid PVAT density in anterior circulation LVO patients was associated with unsuccessful recanalization, poor functional outcome, and increased mortality after MT [15]. In their cohort, PVAT density showed AUC values of approximately 0.70 for these outcomes and remained significant in adjusted models. In contrast, in our cohort, mean ipsilateral PVAT density did not differ by recanalization status or 90-day functional outcome, and effect sizes were small with confidence intervals close to the null. In multivariable models adjusted for age, baseline NIHSS, ASPECTS, onset-to-groin puncture time, and atrial fibrillation, PVAT density was not an independent predictor of either successful recanalization or poor functional outcome. Similarly, ipsilateral PVAT density was not associated with 90-day mortality in univariable logistic regression (OR per 1-HU increase 0.996; *p* = 0.756). Several factors may explain this divergence. First, we restricted inclusion to isolated MCA-M1 occlusions, whereas Jin et al. included a broader anterior LVO spectrum with a substantial proportion of ICA occlusions (38.8%). Second, differences in treatment patterns, clot composition, and collateral status may modulate the impact of pericarotid inflammation on reperfusion and recovery. Third, the primary effect of PVAT density, as suggested by our results, may be largely localized to identifying the underlying LAA stroke rather than predicting the success of MT or longer-term neurological recovery after reperfusion.

The noninvasive quantification of carotid PVAT density provides an easily obtainable, quantitative, and reproducible metric for local carotid vascular inflammation. Given its observed association with LAA stroke, this marker may provide incremental value during acute etiologic assessment, particularly when conventional classification remains uncertain [21]. However, the modest discriminative performance of carotid PVAT density (AUC = 0.67) for identifying LAA stroke warrants cautious clinical interpretation. Accordingly, PVAT density should be interpreted as an adjunctive imaging biomarker rather than an isolated determinant of stroke mechanism. Its potential clinical value may lie in complementing other imaging and clinical indicators of LAA stroke, such as carotid plaque morphology, degree of stenosis, and vascular risk profile [14,22]. In this context, PVAT density may provide additional information on plaque vulnerability beyond luminal narrowing alone, thereby contributing to a more refined etiologic assessment.

Future research should validate these findings in larger, prospective, multi-center MT cohorts and explore advanced PVAT metrics, including volumetric and radiomics-based approaches, which may better capture spatial heterogeneity than small two-dimensional ROIs [4,23]. Combining PVAT density with other CTA features, such as nonstenotic plaque burden, ulceration, and intraluminal thrombus characteristics, may improve etiologic classification [24,25]. Longitudinal studies are also needed to determine whether medical therapies modify PVAT density over time and whether such changes are associated with recurrent ischemic risk [26].

This study has several limitations. First, it was a retrospective, single-center analysis, which introduces the risk of selection bias and limits generalizability. Second, our cohort focused only on isolated MCA-M1 occlusions, limiting the generalizability of outcome findings to ICA or M2 occlusions and to patients who were not eligible for thrombectomy. Third, PVAT density may be influenced by local artifacts and ROI placement, particularly adjacent to heavily calcified plaques. While the reproducibility of the PVAT density measurement technique was high, it relied on manual ROI placement at a single axial level per artery rather than volumetric quantification, and we did not include automated or histogram-based methods. Fourth, CT-based fat attenuation is influenced by acquisition parameters such as tube voltage and reconstruction kernel; our results, obtained on a single scanner with relatively uniform protocol, may not directly translate to other CT systems or settings. Fifth, the number of patients with unsuccessful recanalization was modest, so multivariable models for this outcome may still be prone to overfitting despite the limited covariate set, and small associations cannot be entirely excluded. Finally, we did not have histopathologic confirmation of carotid plaque inflammation and used PVAT density as an imaging surrogate.

## 5. Conclusions

In patients with acute MCA occlusion undergoing mechanical thrombectomy, increased ipsilateral carotid PVAT density was associated with large artery atherosclerotic stroke etiology, suggesting its potential as an adjunctive imaging marker of local carotid inflammation. However, PVAT density was not independently associated with recanalization success or 90-day clinical outcome. Integrating PVAT density with established imaging and clinical markers may refine etiologic classification and risk stratification in the acute setting.

## Figures and Tables

**Table 1:** Baseline characteristics.

Variable	Value
Age, years	72.21 ± 12.39
Male sex, *n* (%)	82 (56.2)
Vascular risk factors, *n* (%)	
Hypertension	92 (63.0)
Diabetes mellitus	40 (27.4)
Dyslipidemia	14 (9.6)
Coronary artery disease	51 (34.9)
Atrial fibrillation	73 (50.0)
Smoking	36 (24.7)
Stroke severity and imaging	
Baseline NIHSS	12.81 ± 4.78
Baseline ASPECTS	8.21 ± 1.44
Acute therapy and procedural data, *n* (%)	
Aspiration technique	23 (15.8)
Combined technique	123 (84.2)
Successful recanalization (mTICI ≥ 2b)	122 (83.6)
Carotid stent/balloon use	15 (10.3)
3-month outcome, *n* (%)	
Poor outcome (mRS > 2)	76 (52.1)
Favorable outcome (mRS ≤ 2)	70 (47.9)
TOAST etiology, *n* (%)	
LAA	38 (26.0)
CE	58 (39.7)
OD/UD	50 (34.2)

NIHSS, National Institutes of Health Stroke Scale; ASPECTS, Alberta Stroke Program Early CT Score; mTICI, modified Thrombolysis in Cerebral Infarction; mRS, modified Rankin Scale; LAA, large-artery atherosclerosis; CE, cardioembolism; OD/UD, other/undetermined etiology.

**Table 2:** Carotid perivascular adipose tissue (PVAT) density and main statistical results.

Analysis	Groups	PVAT (HU), Mean ± SD/Ipsilateral PVAT (HU)	*p* Value
Paired comparison (LAA subgroup)	Ipsilateral (*n* = 38)	−64.24 ± 11.74	<0.001
	Contralateral (*n* = 38)	−78.22 ± 9.13	
TOAST etiology	LAA	−64.24 ± 11.74	
	CE	−75.42 ± 20.11	0.004
	OD/UD	−73.78 ± 14.93	
Post hoc Bonferroni	LAA vs. CE	Δ = 11.19 HU	0.003
	LAA vs. OD/UD	Δ = 9.54 HU	0.004
Recanalization success	Final mTICI < 2b (*n* = 24)	−75.96 ± 16.87	
	Final mTICI ≥ 2b (*n* = 122)	−71.16 ± 17.06	0.21
3-month outcome	mRS > 2 (*n* = 76)	−71.97 ± 17.84	
	mRS ≤ 2 (*n* = 70)	−71.92 ± 16.31	0.99

PVAT, perivascular adipose tissue; HU, Hounsfield unit; LAA, large-artery atherosclerosis; CE, cardioembolism; OD/UD, other/undetermined etiology; TOAST, Trial of Org 10172 in Acute Stroke Treatment; mTICI, modified Thrombolysis in Cerebral Infarction; mRS, modified Rankin Scale; SD, standard deviation.

**Table 3:** Association between ipsilateral PVAT density and LAA etiology (LAA vs. non-LAA).

Model	PVAT OR per 1 HU (95% CI)	*p*-Value	PVAT OR per 5 HU (95% CI)
Univariable	1.042 (1.016–1.070)	0.0017	1.23 (1.08–1.40)
Clinically adjusted multivariable *	1.048 (1.018–1.079)	0.0016	1.27 (1.09–1.46)

* Adjusted for age, sex, atrial fibrillation, hypertension, diabetes mellitus, smoking, and dyslipidemia. Non-LAA is defined as CE + OD/UD.

## Data Availability

The original contributions presented in this study are included in the article. Further inquiries can be directed to the corresponding author.

## Supplementary Materials

The following supporting information can be downloaded at: https://www.mdpi.com/article/10.3390/jcm15114369/s1, Table S1: Correlation between ipsilateral PVAT density and ipsilateral NASCET stenosis severity.

## Abbreviations

The following abbreviations are used in this manuscript:

PVAT	Perivascular adipose tissue
CTA	Computed tomography angiography
MT	Mechanical thrombectomy
MCA	Middle cerebral artery
ICA	Internal carotid artery
TOAST	Trial of ORG 10172 in Acute Stroke Treatment
LAA	Large-artery atherosclerosis
CE	Cardioembolism
OD/UD	Other/undetermined
ROI	Region of interest
HU	Hounsfield unit
ROC	Receiver operating characteristic
AUC	Area under the curve
CI	Confidence interval
OR	Odds ratio
mTICI	Modified Thrombolysis in Cerebral Infarction
mRS	Modified Rankin Scale
ICC	Intraclass correlation coefficient
