# Supplementary material for: Carotid Perivascular Adipose Tissue Density as a Marker of Large Artery Atherosclerotic Stroke in Patients Undergoing Mechanical Thrombectomy for Acute Middle Cerebral Artery Occlusion
Source: J Clin Med. 2026 Jun 5;15(11):4369. doi: 10.3390/jcm15114369 (PMC13258051; doi:10.3390/jcm15114369)
Supplement: Supplementary file 1 [file jcm-15-04369-s001.zip › jcm-4248509-supplementary.pdf]

Table S1. Correlation between ipsilateral PVAT density and ipsilateral NASCET stenosis severity.

Cohort	Pearson r (p)	Spearman ϱ (p)
Overall (n=146)	0.230 (0.0053)	0.165 (0.046)
LAA (n=38)	−0.051 (0.763)	−0.118 (0.481)
non-LAA (n=108)	0.00 (0.997)	−0.045 (0.644)

Ipsilateral NASCET stenosis refers to the cervical ICA on the stroke side; non-LAA includes CE and OD/UD etiologies.
